# Downstream Regulatory Network of MYBL2 Mediating Its Oncogenic Role in Melanoma

**DOI:** 10.3389/fonc.2022.816070

**Published:** 2022-05-18

**Authors:** Feiliang Zhong, Jia Liu, Chang Gao, Tingting Chen, Bo Li

**Affiliations:** ^1^Frontiers Science Center for Synthetic Biology and Key Laboratory of Systems Bioengineering (Ministry of Education), School of Chemical Engineering and Technology, Tianjin University, Tianjin, China; ^2^Collaborative Innovation Center of Chemical Science and Engineering (Tianjin), Tianjin University, Tianjin, China; ^3^Life Science Institute, Jinzhou Medical University, Jinzhou, China; ^4^School of Basic Medicine, Guangdong Medical University, Dongguan, China

**Keywords:** melanoma, MYBL2, ChIP-seq, regulatory network, prognosis

## Abstract

The transcription factor MYBL2 is widely expressed in proliferating cells. Aberrant expression of MYBL2 contributes to tumor malignancy and is associated with poor patient prognosis. However, the downstream transcriptional network that mediates its oncogenic properties remains elusive. In the present study, we observed that MYBL2 was overexpressed in malignant and metastatic melanoma patient samples and that the high expression level of MYBL2 was significantly associated with poor prognosis. A loss-of-function study demonstrated that MYBL2 depletion significantly decreased cell proliferation and migration and prevented cell cycle progression. We also determined that MYBL2 promoted the formation of melanoma stem-like cell populations, indicating its potential as a therapeutic target for treating resistant melanoma. Mechanistically, we constructed an MYBL2 regulatory network in melanoma by integrating RNA-seq and ChIP-seq data. EPPK1, PDE3A, and FCGR2A were identified as three core target genes of MYBL2. Importantly, multivariate Cox regression and survival curve analysis revealed that PDE3A and EPPK1 were negatively correlated with melanoma patient survival; however, FCGR2A was positively correlated with patient survival. Overall, our findings elucidate an MYBL2 regulatory network related to cell proliferation and cancer development in melanoma, suggesting that MYBL2 may be potentially targeted for melanoma diagnosis and treatment.

## Introduction

MYB proto-oncogene like 2 (MYBL2, B-MYB), a member of the MYB transcription factor (TF) family, is widely expressed in most proliferating cells and has a wide range of functions (1). It participates in cell cycle regulation, DNA replication, and maintenance of genome integrity (2–4), suggesting that MYBL2 may be a potential key biomarker. In the cell cycle, the transcription level of MYBL2 can be regulated in an E2F-dependent manner (5). The DREAM complex structure (DP, RB-like, E2F, and MuvB) inhibits cell cycle-related gene expression during the quiescent phase. As cells enter the cell cycle, the MuvB core component of the DREAM complex and FOXM1 cooperate with MYBL2 to co-regulate the expression of G2/M genes (6). MYBL2 is upregulated disproportionately in *p53* gene-mutated tumors, and it can even overcome DNA damage-induced G2 arrest in *p53*-mutated cells (7). The downregulation of MYBL2 leads to cell cycle arrest in the G2/M phase through the p53-p21-DREAM-CDE/CHR pathway (8). MYBL2 participates in different aspects of cell apoptosis and survival by regulating downstream gene and protein interactions. Grassilli et al. demonstrated that MYBL2 regulates anti-apoptotic *Bcl-2* gene upregulation in mouse IL-2-dependent T cells, thus antagonizing doxorubicin-induced apoptosis (9). Moreover, Seong et al. showed that MYBL2 directly interacts with serine–threonine kinase receptor-associated protein (STRAP), so that more tumor suppressor protein TP53 can be translocated to promote cell apoptosis (10). MYBL2 is upregulated in many cancers, such as breast cancer, hepatocellular carcinoma, lung cancer, and colorectal cancer, and upregulated expression of MYBL2 is associated with poor prognosis in patients with cancer. However, additional agents involved in the MYBL2 downstream transcriptional network mediating its cancer-promoting properties remain unclear; furthermore, it is unknown which additional cancer entities are also affected by MYBL2 deregulation (1).

Malignant melanoma (MM) is one of the most aggressive skin tumors originating from melanocytes (11). Although it accounts for only a small number of skin cancers, it is more prone to spread and metastasis; hence, it is the most lethal type of skin cancer (12). Approximately 1.7% of new global cases and 0.6% of new cancer deaths worldwide in 2020 were due to MM (13). In recent years, the discovery of MAPKs and other key signaling pathways, BRAF and other drug targets, and progress in immunotherapy have greatly improved the prognosis of melanoma patients (14). However, due to the strong heterogeneity of melanoma in terms of genetic and epigenetic characteristics, signal transduction pathway activation, and biological behavior, these treatments are still ineffective or suboptimal in a considerable proportion of patients. Cancer stem cells are another major issue for melanoma metastasis and relapse—a small subset of cancer cells can survive and colonize new environments. Therefore, it is essential to develop new and effective approaches targeting cancer stem cells to overcome metastasis and drug resistance in patients with advanced melanoma.

In the present study, we aimed to determine the oncogenic role of MYBL2 and characterize MYBL2-mediated regulatory networks/direct targets in melanoma. Our results indicated that MYBL2 was highly expressed in melanoma samples, revealing a poor prognosis in patients with melanoma. Moreover, we determined that MYBL2 promoted the growth of melanoma cells and melanoma stem-like cell proliferation in a mouse model and in melanoma cells, indicating that MYBL2 may be used as a biomarker or therapeutic target. Next, 11 core target genes of MYBL2 were identified by integrating RNA-seq and ChIP-seq data, suggesting that MYBL2 promoted melanoma growth. Importantly, we identified three key genes (*FCGR2A, PDE3A*, and *EPPK1*) that were correlated with the survival of melanoma patients. These results revealed a MYBL2 regulatory network related to cell proliferation and cancer development pathways in melanoma. MYBL2 may be a potential target for the diagnosis and treatment of melanoma.

## Materials and Methods

### Cell Lines and Cell Culture

Human epidermal melanocyte HEMn-LP, embryonic kidney cell line 293T, and human malignant melanoma cell lines A375 and SK-MEL-28 were purchased from the Cell Resource Center of Peking Union Medical College (IBMS, CAMS/PUMC). The human metastatic melanoma cell line A2058 was kindly provided by Dr. Fang from the Beijing Institute of Genomics. HEMn-LP cells were cultured in 254 medium (Gibco, Thermo Fisher Scientific, Inc.), containing Human Melanocyte Growth Supplement (HMGS, Gibco, Thermo Fisher Scientific, Inc.), 100 μg/ml penicillin, and 100 μg/ml streptomycin. 293T, A375, SK-MEL-28, and A2058 cell lines were cultured in Dulbecco’s modified Eagle’s medium (DMEM) supplemented with 10% fetal bovine serum (FBS) (Gibco, Thermo Fisher Scientific, Inc.), 100 μg/ml penicillin, and 100 μg/ml streptomycin. The cultures were maintained in a humidified incubator with 5% CO_2_ at 37°C under standard cell culture conditions and routinely passaged when 80%–90% confluent.

### Data Collection and Bioinformatic Analysis

Melanoma transcriptome data were obtained from The Cancer Genome Atlas (TCGA) database (https://portal.gdc.cancer.gov). Normal skin samples were obtained from the GTEx database. mRNA expression data involving 461 tumors and 558 normal patient samples were collected. For Kaplan–Meier curves, *p*-values and hazard ratios (HRs) with 95% confidence intervals (CIs) were generated by log-rank tests and univariate Cox proportional hazard regression. All analytical methods indicated above and R packages were performed using R software v.4.0.3 (15). *p* < 0.05 was considered to be statistically significant.

### Tissue Microarrays and Immunohistochemistry

Skin cancer tissue microarray (TMA) (K063Me01) was purchased from Xi’an Biotech Co., Ltd. (Xi’an, China). Protein expression was detected by immunohistochemistry (IHC) and analyzed according to standard methods and microarray instructions. IHC staining was performed with a specific antibody (MYBL2, Thermo Fisher Scientific, PA5-79713) and then TMAs were examined and independently scored by two pathologists. Tumor stages of the specimens on the TMA were categorized according to the tumor–node–metastasis (TNM) system of the American Joint Committee on Cancer (AJCC) (16). Negative control (NC) groups were examined using conventional hematoxylin and eosin (H&E) staining. H&E staining was performed according to standard methods. IHC experimental evaluation criteria: After locating the staining results on the chip point by point, the color intensity of the cells was judged as follows: no staining = negative (-), light brown = weakly positive (+), brown = positive (++), and Tan = strongly positive (+++). According to the number of positive cells, subdivision into (-) means that the number of positive cells = <10%, (+) means that the number of positive cells = 10%–25%, (++) means that the number of positive cells is between 26% and 49%, and (+++) means that the number of positive cells = >50%. Finally, a qualitative and semi-quantitative color intensity result was obtained based on a comprehensive evaluation of the two results. At least 5–10 HPFs (high-power fields) were randomly observed, and average values were calculated.

### *MYBL2* Silencing and Overexpression

Pairs of complementary oligonucleotides encoding shRNAs were cloned into the lentiviral mammalian expression vector pLL3.7 (Addgene, Watertown, USA) according to the manufacturer’s instructions. The target sequences of the shRNA were as follows: sh1, 5′-GCTAACAACAAAGTTCCACTT-3′, and sh2, 5′-GCTTGGTGTGACCTGAGTAAA-3′. A non-silencing shRNA sequence without the *MYBL2* shRNA component was used as an NC. For infection, 5 × 10^5^ 293T cells were plated in 6-cm plates and transfected 24 h later with 1 μg of DNA from lentiviral backbone vector and packaging plasmids according to the Lipofectamine 3000 transfection kit (Invitrogen, Thermo Fisher Scientific, Inc.) protocol. The medium was replaced with DMEM 24 h post-transfection. Cells were infected for 24 h at 37°C with 2 ml of lentivirus and 8 μg/ml polybrene (Sigma-Aldrich).

Full-length cDNA encoding human MYBL2 was synthesized and inserted into the pCDH-CMV-GFP-T2A-Puro vector (Addgene) to obtain the MYBL2-overexpressing plasmid pCDH-MYBL2. The recombinant lentiviral vector pCDH-MYBL2 was then transfected into melanoma A375 and A2058 cells. The transfection reagent Lipofectamine 3000 (Invitrogen) was mixed with Opti-MEM (Gibco, Thermo Fisher Scientific). Cells were infected for 24 h at 37°C with 2 ml of lentivirus and 8 μg/ml polybrene (Sigma-Aldrich). Cells were selected 48 h later using 1 μg/ml puromycin (Sigma-Aldrich). Knockdown and overexpression efficiency were determined by qPCR of *MYBL2* mRNA and Western blot assays for MYBL2 protein.

### Quantitative Real-Time Polymerase Chain Reaction

Total RNA was extracted from cultured cells using TRIzol reagent (Invitrogen, Thermo Fisher Scientific, Inc.) and cDNA was reverse-transcribed using the GeneCopoeia™ First Strand cDNA Synthesis Kit (Genecopoeia, USA). RT-qPCR analysis was performed using the SYBR PCR mix kit (TransGen, Beijing, China) according to the manufacturer’s instructions. The samples were run in triplicate in three independent experiments. *GAPDH* RNA was used as a reference housekeeping gene. All primer sequences were designed using Primer v.5.0 software (Premier Biosoft International, Palo Alto, CA, USA) as follows:

homo *MYBL2* forward, 5′-GTCCCCTGTCACTGAGAATAG-3′;
homo *MYBL2* reverse, 5′-GCTCCAATGTGTCCTGTTTG-3′;
homo *GAPDH* forward, 5′-AGCCACATCGCTCAGACAC-3′;
homo *GAPDH* reverse, 5′-TTAAAAGCAGCCCTGGTGAC-3′.

Transcript levels were calculated using the comparative threshold cycle (Ct) method normalized to *GAPDH* abundance.

### Western Blotting

Western blot analysis was performed according to standard protocols. PVDF membranes (Bio-Rad, Hercules, CA, USA) were probed with specific antibodies, and immunoreactive proteins were detected using an enhanced chemiluminescence (ECL) kit (Thermo Fisher Scientific). GAPDH served as an internal control and was imaged and analyzed using a C-Digit Blotting Scanner (Azure Biosystems, Inc.). Human anti-MYBL2 antibody was obtained from Thermo Fisher Scientific (PA5-79713).

### Cell Proliferation Assay

Inhibition of cell proliferation was quantified by Cell Counting Kit-8 (CCK-8; TransGen, Beijing, China) following the manufacturer’s instructions. Ten microliters of CCK-8 kit solution was added to the medium after a total of 3 × 10^3^ cells were seeded into each well of 96-well plates. The optical density (OD) was measured at 450 nm using a microplate reader (Thermo Fisher Scientific, Inc.). Each measurement was repeated three times.

### Flow Cytometric Analysis

Flow cytometric analysis was performed to determine the effect of MYBL2 on cell cycle distribution. Briefly, 3×10^5^ cells grown in 6-well plates were treated with shRNA for 48 h. The cells were then harvested and fixed in 75% ethanol solution. After centrifugation, cells were washed (PI) for 30 min in the dark. Cell cycle distribution was analyzed by flow cytometry (NovoCyte 2040R; ACEA Bioscience, Inc.; Agilent Technologies).

### Wound Healing and Transwell Migration Assays

Cell migration ability was assessed by wound healing and transwell migration assays. In the wound healing assay, in brief, 5 × 10^5^ cells were cultured in 6-well plates in DMEM supplemented with 10% FBS to 80%–90% confluence in 24 h. The plate was scratched using a sterile 10-μl pipette tip to generate a uniform wound in the cell monolayer. The plate was washed with PBS to remove cell debris. After continuous incubation for 24 h, wound closure was monitored using an inverted fluorescent microscope. The width of the wound gap was analyzed using ImageJ software. The wound closure area was calculated as follows: migration area (%) = (A_0_ − An)/A_0_ × 100, where A_0_ represents the area of the initial wound area, and An represents the remaining area of the wound at the metering point.

In the transwell migration assay, cells were collected and seeded into the upper chamber (8 µm) at a density of 1 × 10^5^ cells/well (Corning Inc., Corning, NY, USA). The lower chamber was filled with 800 µl of DMEM supplemented with 10% FBS, and the cells were incubated for 24 h at 37°C. The lower cells were fixed with 4% (w/v) formaldehyde and stained with 0.1% (w/v) crystal violet for 30 min. The number of migrated cells was counted under a microscope.

### Stemness Indices Calculation

From the TCGA database, we downloaded RNA-seq (FPKM, Fragments Per Kilobase per Million) of melanoma cases from the Genomic Data Commons (GDC). Next, we converted the PFKM data to TPM and normalized the data log2 (TPM+1) while keeping samples with clinical information recorded. We then calculated mRNA stemness indices using the OCLR algorithm constructed by Malta et al. (17). Based on the characteristics of mRNA expression, the gene expression profile contained 11,774 genes. We used the same Spearman correlation (RNA expression data) and then subtracted the minimum value and divided the difference by the linear transformation of the maximum value to map the dryness index to the range [0,1]. These analysis methods and R package were implemented by R Foundation for Statistical Computing (15) v.4.0.3.

### Colony Formation Assay

For tumorsphere formation, single-cell suspensions were harvested and seeded into 6-well ultra-low adherent cell culture plates at a density of 1,000 cells/ml in serum-free DMEM/F12 medium supplemented with 1% L-glutamine, 1% penicillin/streptomycin, 2% B27 (Invitrogen), 20 ng/ml epidermal growth factor (EGF, Sigma, St. Louis, MO, USA), and 20 ng/ml basic fibroblast growth factor (bFGF, Invitrogen). Seven days after seeding, tumorspheres with diameters > 30 μm were counted using Olympus cellSens Standard software. The total numbers of tumorspheres in 6 random fields under 10× objective lens were determined for each well. The experiments were repeated at least 3 times.

### *In Vivo* Tumorigenicity

Six- to eight-week-old male BALB/c-nu/nu mice were purchased from Biotechnology Co., Ltd. (Beijing, China). Cells (1 × 10^7^) in 100 μl of PBS were injected subcutaneously into the right flank of the mice. Body weight was monitored twice per week. Tumor dimensions were measured using calipers, and tumor volume size was calculated using the equation (length × width^2^/2). At the end of the experiment, the mice were euthanized, and the tumors were weighed and processed for further analysis. All animal experiments were performed in accordance with protocols approved by the Animal Ethics and Welfare Committee (AEWC) (approval no. IRM-DWLL-2019102).

### Histology and Morphometric Analysis

Tumors were collected and fixed in 10% neutral-buffered formalin. Tissues were sectioned and stained with H&E. Images were acquired using an optical microscope (BX51, Olympus, Tokyo, Japan) to evaluate pathological morphology.

### Whole-Transcriptome Sequencing

Total RNA was extracted and lysed in 500 μl of TRIzol reagent (MRC, Carrollton, OH, USA) and sent to China’s Shenzhen BGI (Shenzhen, China) for further analysis. An RNA-Seq library was created using the Illumina TruSeq RNA Sample Preparation Kit v.2 using a standard protocol. Genes with a *p*-adjusted value (false discovery rate) < 0.05 were selected for Gene Ontology (GO) analysis and heatmap construction. Pathway analysis was performed using the Kyoto Encyclopedia of Genes and Genomes (KEGG) database.

### Chromatin Immunoprecipitation Followed by Gene Sequencing assay

ChIP assays were performed using the SimpleChip Plus Sonication Chromatin IP Kit (Cell Signaling Technology, China) following the manufacturer’s instructions. Briefly, A2058 cells were cross-linked with 1% formaldehyde solution for 10 min at room temperature and lysed in ChIP lysis buffer with freshly added 1× protease inhibitor cocktail (Roche Applied Science). Cross-linked DNA was then sheared to ~200- to 700-bp fragments *via* sonication with the following pulse mode settings: 10 s with 50 s cooling, amplitude 30%, and 8 cycles. Chromatin was then immunoprecipitated with pMYBL2 antibody (Abcam, ab76009) and DNA was recovered after phenol/chloroform extraction and ethanol precipitation. High-throughput sequencing using an Illumina HiSeq 3000 Sequencer was performed by the Chinese Shenzhen-based BGI (Shenzhen, China).

### Statistics

Statistical analyses were performed using Prism 8 software (GraphPad, La Jolla, CA, USA). For comparisons between two groups, two-tailed Student’s *t*-test was used. Kaplan–Meier survival analysis was performed to compare survival curves. The statistical significance of protein associations in the TMA dataset was evaluated using Pearson’s chi-squared test. Statistically significant levels were defined as ns (not significant, *p* > 0.05), * *p* < 0.05, ** p < 0.01, ***p < 0.001. All data are presented as means ± SD.

## Results

### MYBL2 Is Upregulated in Patients With Melanoma

**Figure 1:**
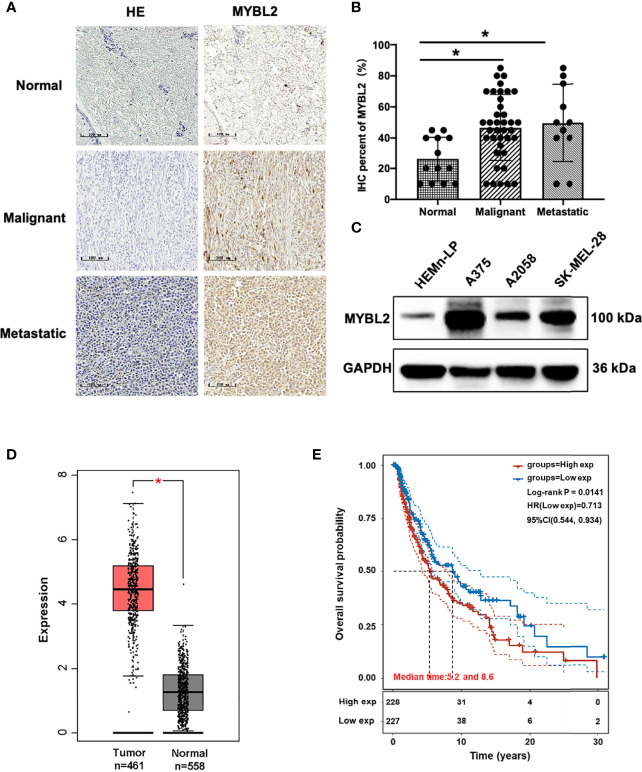
MYBL2 is upregulated in patients with melanoma. **(A)** IHC was performed to detect the expression of MYBL2 in normal skin tissue, malignant melanoma, and metastatic malignant melanoma samples (×200). **(B)** Analysis of IHC showing MYBL2 expression. **(C)** Western blot analysis of MYBL2 in three distinct cell lines: normal stage (HEMn-LP), onset of malignant melanoma (A375 and SK-MEL-28), and metastatic stage (A2058). **(D)** MYBL2 expression in 558 normal human and 461 melanoma patients. Analysis of *MYBL2* mRNA expression across various types of samples based on the melanoma dataset from The Cancer Genome Atlas Genomic Commons (TCGA-GDC) Data Portal using GEPIA interactive web server. Normal skin sample data were obtained from the GTEx database. **(E)** High MYBL2 expression level correlated with poor survival of melanoma patients. Kaplan–Meier curve analysis based on the TCGA Skin Cutaneous Melanoma (SKCM) dataset showing melanoma patient overall survival grouped by high *MYBL2* mRNA expression level (upper quartile, *n* = 120) versus those with low *MYBL2* expression level (lower quartile, *n* = 120). *p < 0.05.

To determine the role of MYBL2 in human melanoma, paraffin sections of skin cancer TMAs (K063Me01) were stained by IHC. The results showed that MYBL2 protein was highly expressed in tumor cells, and a brown granular distribution was observed in the cells and cytoplasm. In the control group, the expression level of MYBL2 was low; in the malignant melanoma group, the degree of MYBL2 IHC staining was significantly increased, and most samples exhibited moderate positivity. In the metastatic melanoma group, the degree was the deepest, and most samples showed strong positivity (**Figures 1A, B**). Detailed clinical and pathological information are shown in **Table 1**. These results coincided with publicly available datasets of melanoma patients recorded from TCGA using the GEPIA interactive web server (18), which demonstrated that MYBL2 is significantly upregulated in malignant melanoma tissues compared with normal tissues (**Figure 1D**). The expression of MYBL2 was also detected in human melanocytes (HEMn-LP), human malignant melanoma cell lines (A375 and SK-MEL-28), and metastatic melanoma cell lines (A2058) by Western blotting. MYBL2 was expressed in all the tested cell lines, and higher expression level was observed in melanoma cell lines (A375, SK-MEL-28, and A2058) than in melanocytes (HEMn-LP) (**Figure 1C**).

**Table 1:** Clinical–pathological information and TNM staging of human melanoma specimens (*n* = 63) used in this study.

Subject	Location	Type	Subject	Location	Type
A1	Skin	Malignant	B1	Skin	Malignant
A2	Skin	Malignant	B2	Skin	Malignant
A3	Skin	Malignant	B3	Skin	Malignant
A4	Skin	Malignant	B4	Skin	Malignant
A5	Skin	Malignant	B5	Skin	Malignant
A6	Skin	Malignant	B6	Skin	Malignant
A7	Skin	Malignant	B7	Skin	Malignant
A8	Skin	Malignant	B8	Skin	Malignant
C1	Skin	Malignant	D1	Skin	Malignant
C2	Skin	Malignant	D2	Skin	Malignant
C3	Skin	Malignant	D3	Skin	Malignant
C4	Skin	Malignant	D4	Skin	Malignant
C5	Skin	Malignant	D5	Skin	Malignant
C6	Skin	Malignant	D6	Skin	Malignant
C7	Skin	Malignant	D7	Esophagus	Malignant
C8	Skin	Malignant	D8	Urethra	Malignant
E1	Cavidade nasal	Malignant	F1	Lymph node	Metastasis
E2	Cavidade nasal	Malignant	F2	Lymph node	Metastasis
E3	Mediastinum	Malignant	F3	Lymph node	Metastasis
E4	Skin	Malignant	F4	Lymph node	Metastasis
E5	Skin	Malignant	F5	Small intestine	Metastasis
E6	Eye	Malignant	F6	Lymph node	Metastasis
E7	Lymph node	Metastasis	F7	Liver	Metastasis
E8	Lymph node	Metastasis	F8	Lymph node	Metastasis
G1	Skin	Control	H1	Oral cavity	Control
G2	Skin	Control	H2	Esophagus	Control
G3	Skin	Control	H3	Esophagus	Control
G4	Skin	Control	H4	Small intestine	Control
G5	Skin	Control	H5	Small intestine	Control
G6	Skin	Control	H6	Lymph node	Control
G7	Skin	Control	H7	Lymph node	Control
G8	Oral cavity	Control			

Kaplan–Meier analysis based on TCGA data revealed that high MYBL2 expression level was positively correlated with poorer progression-free survival of melanoma patients in the cohort of cutaneous melanoma (*p* = 0.0141, **Figure 1E**). According to MYBL2 expression levels, 455 melanoma patient samples were allocated into low- and high-MYBL2-expressing groups. The Kaplan–Meier survival plot was grouped by the median MYBL2 expression level in melanoma samples. In conclusion, these results illustrated a strong association between MYBL2 expression level and reduced survival in melanoma patients, and suggested that MYBL2 may be a useful biomarker for patient diagnosis and prognosis in melanoma cases.

### MYBL2 Is Essential for Melanoma Cell Proliferation and Migration

**Figure 2:**
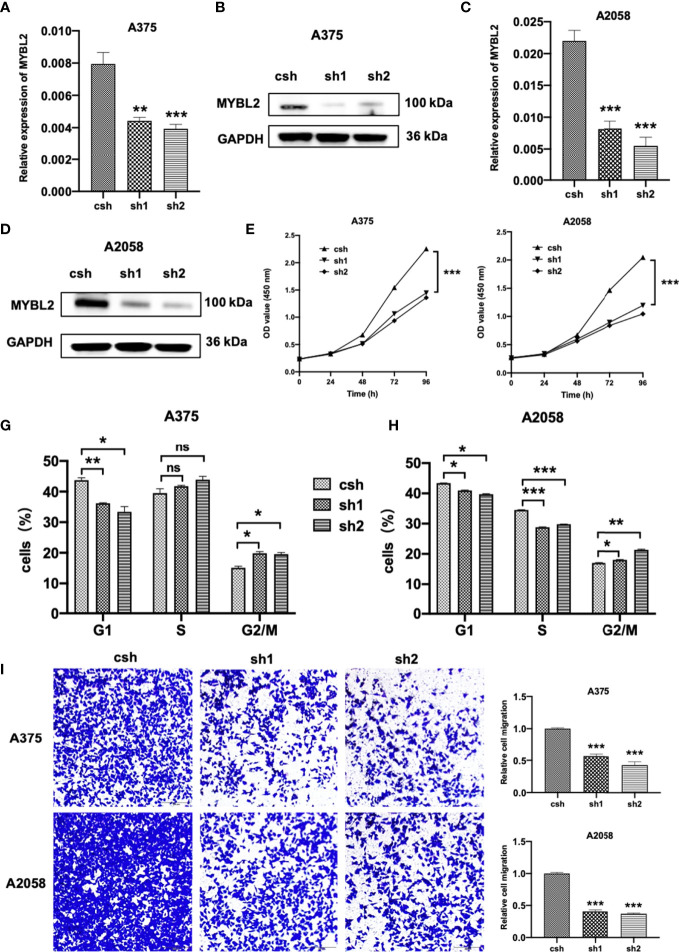
MYBL2 is essential to cell survival in melanoma cells. **(A–D)** qPCR and Western blot analyses of MYBL2 in melanoma cell lines A375 and A2058 using pLL3.7 lentivirus-expressing control shRNA (csh) and 2 different *MYBL2* shRNAs (sh1 and sh2). **(E, F)** Proliferation of cells with csh, sh1, and sh2 targeting *MYBL2* at 0, 24, 48, and 96 h was detected. **(G, H)** Flow cytometric analysis was performed to assess cell cycle phase status after staining with propidium iodide (PI). **(I)** Images and quantitative cell migration of melanoma cells after transfection with *MYBL2*-shRNAs lentiviruses. These experiments were repeated at least 3 times. *p < 0.05, **p < 0.01, ***p < 0.001. ^ns^*P* > 0.05, no significant difference.

To study the effects of MYBL2 on the biological behavior of melanoma cells and its role in tumor formation and growth rate, shRNA was used for gene silencing. The effect of *MYBL2* knockdown (KD) was confirmed by qPCR and Western blotting (**Figures 2A–D**). Silencing of *MYBL2* obviously inhibited the proliferation of melanoma cells compared to the control (**Figures 2E, F**).

The effects of MYBL2 on cell cycle progression were analyzed using flow cytometry. As shown in **Figures 2G, H**, the proportion of cells in the G2/M cell cycle phase was significantly increased, while the proportion of cells in the G1 phase was markedly decreased in A375 and A2058 cells. These data showed that *MYBL2* KD induced G2/M phase arrest.

To detect the relationship between MYBL2 expression and the migration of melanoma cells, wound healing and transwell migration assays were performed. shMYBL2 plasmids, which were transfected into A375 and A2058 cells, inhibited the migratory ability of these cells (**Supplementary Figure 1** and **Figure 2I**). Wound healing and migration rates of sh1 and sh2 cells were significantly lower than those in the control groups. These studies indicated that MYBL2 promoted the proliferation and migration of melanoma cells.

### MYBL2 Promotes Tumor Growth *In Vivo*

**Figure 3:**
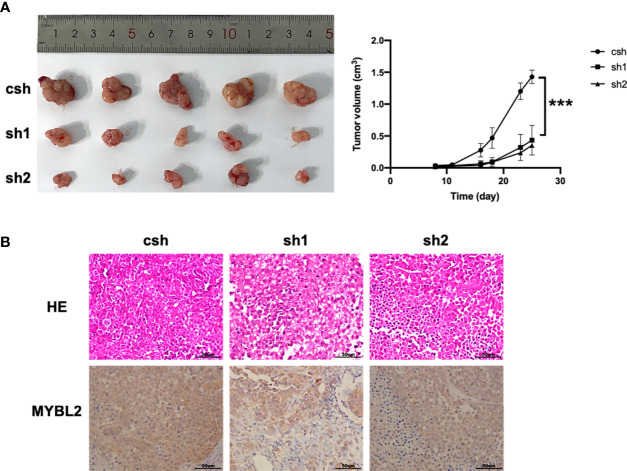
MYBL2 promotes tumor growth and progression in xenograft mice. Twenty-five days after subcutaneous inoculation of melanoma cells, tumors were removed, and diameters were measured (*n* = 5/group) ± standard deviation (SD). **(A)** Subcutaneous tumors generated in BALB/c-nu/nu mice, with *MYBL2* KD-transduced A2058 cells. **(B)** Histological analysis of 25-day-old subcutaneous tumors after H&E and IHC staining for MYBL2. Magnification × 200. ***p < 0.001.

We further explored whether MYBL2 affects melanoma growth *in vivo*. A2058 cells stably transfected with the *MYBL2* shRNA vector were inoculated into male nude mice to observe the effects of *MYBL2* KD on tumor growth and progression. As shown in **Figure 3A**, KD of *MYBL2* in tumor cells strongly inhibited tumor cell growth compared to that in the control group. Consistently, *MYBL2* KD tumors had reduced cell proliferation, and the levels of MYBL2 were significantly decreased in MYBL2 KD tumors compared with the control group (**Figure 3B** and **Supplementary Figure 2**). These findings indicated that MYBL2 can affect melanoma cell growth *in vivo*.

### MYBL2 Promotes the Growth of Melanoma Stem-Like Cell Populations

**Figure 4:**
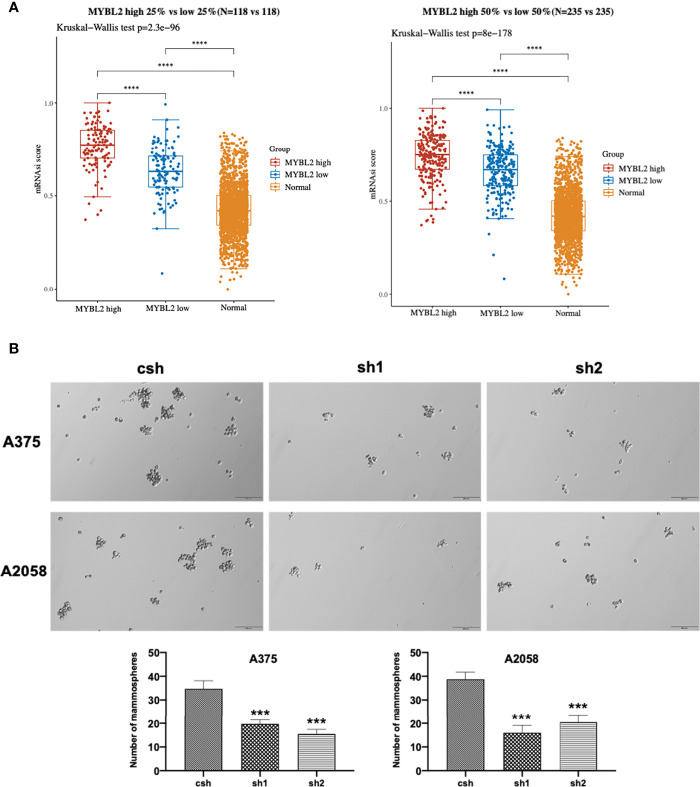
MYBL2 promotes the growth of MSLC populations. **(A)** The distribution of OCLR scores in different groups, where the horizontal axis represents samples of different groups, and the vertical axis represents the distribution of OCLR scores, where different colors represent different groups. The upper left corner represents the significance *p*-value test method. **(B)** Tumorsphere formation in A375 and A2058 expressing csh or *MYBL2* shRNAs (sh1 and sh2). Seven days after seeding, tumorspheres with diameters > 30 μm were counted using Olympus cellSens Standard software. The total numbers of tumorspheres in 6 random fields under 10× objective lens were determined for each well. The experiments were repeated at least 3 times. ****p < 0.0001.

The colony-forming assay is related to stem cell features (19–21). To evaluate the stemness of MYBL2-expressing melanoma cells, we downloaded and converted melanoma RNA-Seq (FPKM) data to TPM and normalized the data log2 (TPM+1), while keeping samples with clinical information recorded. We then calculated the stemness indices (mRNAsi) of high MYBL2 (top 50% and 25%) and low MYBL2 (top 50% and 25%) groups using a one-class logistic regression machine-learning algorithm (OCLR) (**Figure 4A**).

Next, we analyzed the effect of *MYBL2* KD on colony formation in A375 and A2058 cells. As shown in **Figure 4B**, *MYBL2* KD significantly inhibited colony-forming ability compared to the control cells. These results indicated that MYBL2 may play a major role in stem cell homeostasis in MSLCs.

### MYBL2 Resulted In Distinct Genetic Profiling

**Figure 5:**
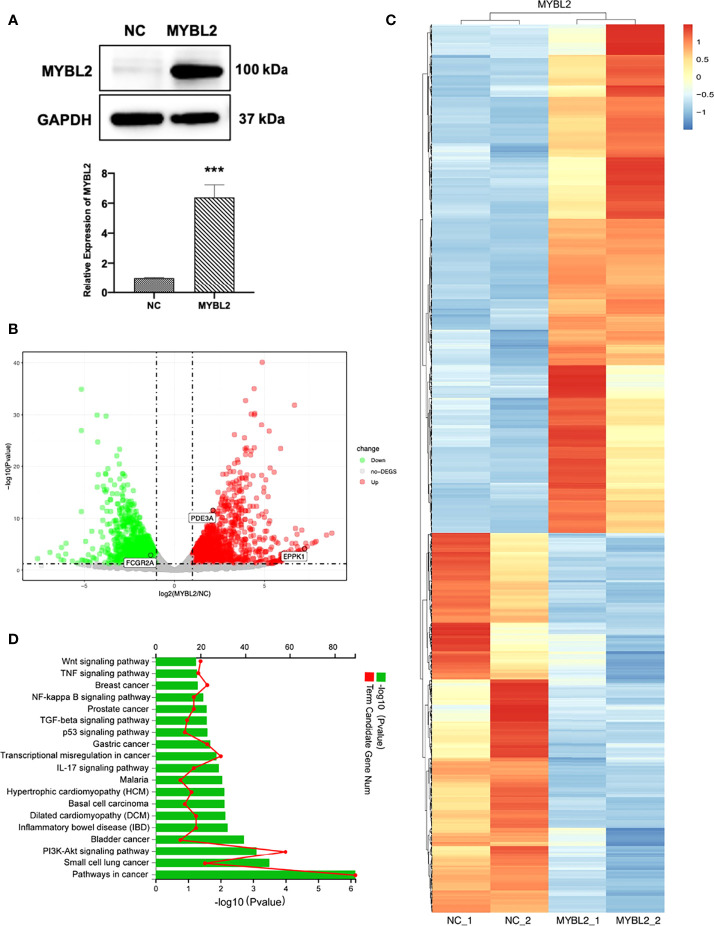
Distinct genetic profiling of MYBL2. **(A)** Western blot of MYBL2 in A2058 cells with control or MYBL2 overexpression. **(B)** Volcano plot showing differential gene expression (1,874 genes, FDR-corrected *p*-value < 0.05) in A2058 cells after overexpression of MYBL2. With ≥2-fold change cutoff, 810 genes were relatively downregulated and 1,064 genes were upregulated. **(C)** Heatmap of A2058 cells with stable MYBL2 overexpression. **(D)** KEGG pathway enrichment analysis (representative pathways) of genes in A2058 cells with stable MYBL2 overexpression. ***p < 0.001.

The introduction of *MYBL2* into A2058 cells was confirmed by qPCR and Western blotting (**Figure 5A**). To explore the molecular mechanism of MYBL2 expression in melanoma, whole transcriptome sequencing of MYBL2-overexpressing A2058 cells was performed. Gene expression analysis using volcano plots showed 1,874 differentially expressed genes, including 810 downregulated genes and 1,064 upregulated genes [genes with a fold change ≥ 2 and a *p*-value (Student’s *t*-test) < 0.05] (**Figure 5B**). Moreover, the heatmaps of two replicates of the control and MYBL2-treated samples exhibited highly consistent transcriptional changes (**Figure 5C**). KEGG pathway analysis was performed to detect *MYBL2* gene expression in melanoma cells. The top five pathways following MYBL2 treatment included pathways in cancer, small cell lung cancer, PI3K-Akt signaling pathway, bladder cancer, and inflammatory bowel disease (IBD) (**Figure 5D**).

### Identification of MYBL2 Targets in Melanoma Cells

**Figure 6:**
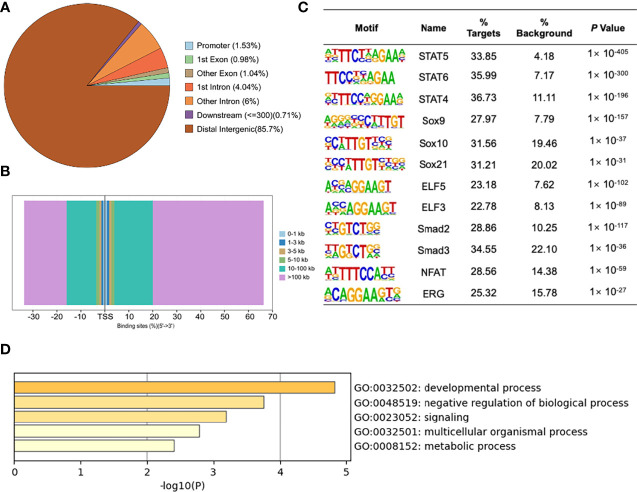
ChIP-Seq profiles of MYBL2 in melanoma cells. **(A)** Pie chart of the percentage of MYBL2-binding sites across different human genomic regions. **(B)** Distribution of MYBL2 binding sites from ±100 kb to the transcriptional start site (TSS) across the human genome (*x*-axis, number of peaks in the genome; *y*-axis, distance relative to the TSS from −100 kb to +100 kb). **(C)** Logos of significantly enriched motifs detected. **(D)** GO enrichment analysis of MYBL2-binding target genes.

To detect the specific transcription factor binding sites (TFBSs) of MYBL2 in A2058 cells, we performed ChIP-Seq to examine the genome-wide distribution of MYBL2 binding sites. The genomic locations of enriched peaks, annotated to the most proximal transcription start site (TSS), exhibited a wide distribution pattern (**Figure 6A**). In total, 85.7% of MYBL2 binding sites were in distal intergenic regions and 1.53% sites were located near gene promoters, while 2.02% and 10.04% mapped to exons and introns, respectively. Binding regions were identified from +100 kb to −100 kb. We detected only a few peaks close to the ± 3 kb TSS, and several peaks were located in intergenic regions >3 kb from the TSS (**Figure 6B**).

Binding site enrichment is a powerful tool for identifying relationships between characterized TFs of genes determined from genome-scale profiling experiments. We used Logos to display the top-scoring predicted motifs sorted based on *p*-values. The enrichment results identified a series of motifs of TFs with signal transducer and activator of transcription (STAT5, STAT6, and STAT4), SRY-box TF (SOX9, SOX10, and SOX21), E74-like ETS TF (ELF3 and ELF5), Smad TFs (Smad2 and Smad3), NFAT, and ERG (**Figure 6C**). Most of the top enriched TFs are involved in cell proliferation and cancer development (22–26), which could lead to abnormal cell proliferation, cell cycle progression, and apoptosis inhibition in many cancers, thereby enhancing the development of tumors (22); SOX family members are widely involved in the development of human malignant tumors (23). We determined that MYBL2 participates in developmental processes, signaling, and multicellular organismal processes by enriching MYBL2 binding target genes (**Figure 6D**).

**Figure 7:**
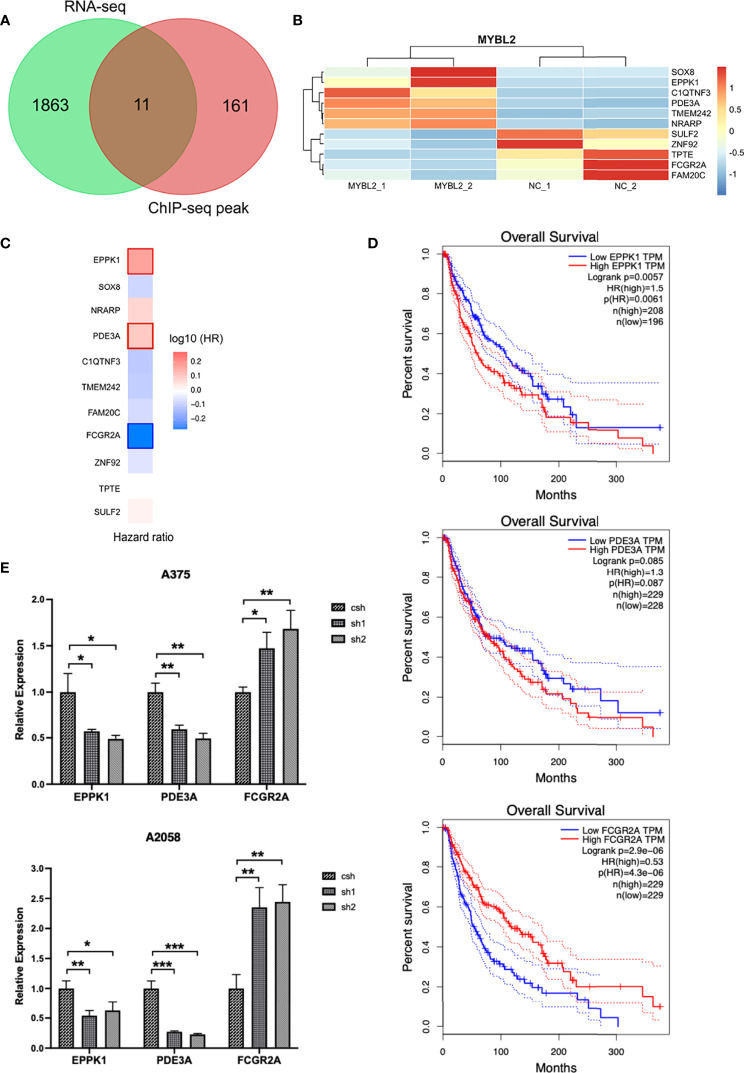
Combined transcriptome profiling and ChIP-Seq analysis identifies 3 highly plausible direct targets of MYBL2. **(A)** Venn diagram showing the overlap of genes among RNA-Seq differentially expressed genes (DEGs) and ChIP-Seq peak. **(B)** Heatmap of 11 significantly DEGs. The data form the transcription profiling of A2058 with overexpressed MYBL2. **(C)** Multivariate Cox regression analysis of 3 significant genes (*EPPK1, PDE3A*, and *FCGR2A*). **(D)** Survival curve of 3 significant differential genes (*EPPK1, PDE3A*, and *FCGR2A*) using Kaplan–Meier curve analysis in melanoma patients. **(E)** Relative expression levels of MYBL2 target genes (*EPPK1, PDE3A*, and *FCGR2A*) in A375 and A2058 cells infected with *MYBL2*‐shRNAs lentiviruses compared with scrambled shRNA lentiviruses. *p < 0.05, **p < 0.01, ***p < 0.001.

MYBL2 target genes revealed the role of MYBL2 in cell proliferation and development. As RNA-Seq and ChIP-Seq are complementary approaches for elucidating gene regulatory mechanisms, we employed a combination of ChIP-Seq and RNA-Seq analysis on a genome-wide level. Integrated ChIP-Seq and RNA-Seq data analysis revealed that there were 11 overlapping genes, including 5 downregulated genes (*SULF2, TPTE, ZNF92, FCGR2A*, and *FAM20C*) and six upregulated genes (*TMEM242, C1QTNF3, PDE3A, NRARP, SOX8*, and *EPPK1*) (**Figures 7A, B**). In univariate Cox proportional hazard regression analysis, 3 of 11 genes (*FCGR2A, PDE3A*, and *EPPK1*) were significantly related to the prognosis of patients with melanoma (**Figure 7C**). To further investigate the prognostic analysis of three genes (*FCGR2A, PDE3A*, and *EPPK1*) in the prognostic model, Kaplan–Meier analysis indicated that three genes were associated with patient survival rate**;** *PDE3A* and *EPPK1* were negatively correlated with survival of melanoma patients; however, *FCGR2A* was positively correlated with melanoma patient survival (**Figure 7D** and **Supplementary Figure 3**). To determine pairwise correlations involving MYBL2 expression and three genes (*FCGR2A, PDE3A*, and *EPPK1*), we reanalyzed the transcriptome data of melanoma cases from TCGA. We determined that MYBL2 expression was positively or negatively correlated with *PDE3A* (**Supplementary Figure 2B**, *R* = 0.1, *p* = 0.03) and *FCGR2A* (**Supplementary Figure 2C**, *R* = −0.15, *p* = 1.23e-03) expression. Next, the levels of *EPPK1, PDE3A*, and *FCGR2A* were determined in A375 and A2058 cells infected with *MYBL2*-shRNA lentiviruses by qPCR (**Figure 7E**). We observed that the levels of *EPPK1* and *PDE3A* diminished, while FCGR2A was upregulated in the *MYBL2*-shRNA group compared with the scrambled shRNA group. In summary, these results revealed that three key genes (*FCGR2A, PDE3A*, and *EPPK1*) may be potential prognostic factors in patients with melanoma.

## Discussion

Melanoma is a malignant invasive tumor, and its global incidence rate is increasing. In the past decade, great progress has been made in elucidating the mechanisms of melanoma occurrence and progression. The treatment of melanoma patients has improved—local melanoma resection and local lymph node dissection, radiotherapy, chemotherapy or natural chemical combination therapy, gene therapy, and immunotherapy can be used to inhibit the metastasis of melanoma *in vitro* and *in vivo*. However, the long-term prognosis of patients with metastatic melanoma remains unsatisfactory, and the underlying mechanism of the pathogenesis and progression of melanoma remains to be elucidated. Therefore, it is important to identify effective molecular markers to explore new therapeutic targets.

MYBL2 is an important TF that mediates the occurrence and development of many types of tumors. It promotes the malignant transformation of tumors by regulating the biological processes of tumor cell proliferation (27), apoptosis (28), migration (29), and invasion (30). It has typical oncogenic characteristics. The difference in mRNA expression levels between cancer tissues and normal tissues is helpful in determining whether gene expression is related to the occurrence of cancer. In this study, the expression and function of MYBL2 in melanoma were studied in clinical cases and *in vivo* and *in vitro.* We observed that the expression level of MYBL2 in melanoma was higher than that in normal skin tissue and was associated with the progression and poor prognosis of melanoma patients. The results of this study are consistent with those of previous reports. Overexpression of MYBL2 is observed in a variety of tumors and is related to poor prognosis. Previous studies have also indicated that *MYBL2* mRNA is overexpressed in cervical cancer using gene expression profiling and TaqMan PCR (31). Ren et al. also confirmed that the expression of MYBL2 is related to the prognosis of colorectal cancer patients. Through Cox multivariate regression analysis of the prognosis of colorectal cancer patients, MYBL2 protein expression and tumor stage were seen to be independent prognostic factors (32). Guan et al. selected cases of primary hepatocellular carcinoma from the TCGA database. Bioinformatic analysis revealed that the expression levels of *MYBL2* mRNA and exon were significantly higher in the death group, and overall patient survival was poorer in the high-expression group of *MYBL2* mRNA and exon. Univariate and multivariate regression analyses confirmed that high expression level of *MYBL2* mRNA was an independent prognostic factor in patients with hepatocellular carcinoma (33).

Further study surrounding the mechanisms involving MYBL2 in melanoma by cytological functional testing is helpful to explain the expression of *MYBL2* in tissues. In a subsequent *in vitro* mechanism study, we determined that shRNA lentivirus-mediated *MYBL2* reduction could inhibit the proliferation, metastasis, and cycle arrest of melanoma cells. MYBL2 can promote cancer progression by promoting tumor cell proliferation and inducing treatment resistance and metastatic diffusion. Ren used siRNA to interfere with the expression of MYBL2 in colon cancer, and the proliferation of tumor cells was decreased (32). Jin et al. also showed that overexpression of MYBL2 can promote the proliferation of non-small cell lung carcinoma (NSCLC) cells, and that the ERK and Akt signaling pathways are involved in the regulation of MYBL2 in NSCLC (34). Other studies have also supported the relationship between MYBL2 and cell proliferation. Cell proliferation is related to the cell cycle; DNA-damaged cells do not progress through the G2/M phase, which leads to an increase in the number of cells arrested in the G2/M phase (35). In our study, we determined that *MYBL2* KD decreased the proportion of cells in the G1 phase and induced G2 phase arrest in human melanoma A375 and A2058 cell lines. These results are consistent with those of previous studies (36–38); however, the underlying mechanisms need to be further explored.

The results of the present study showed that MYBL2 promoted cell proliferation. However, we need to further explore how MYBL2 affects the proliferation of melanoma cells. The integration of ChIP-Seq and RNA-Seq results showed that five genes were downregulated and six genes were upregulated. Moreover, the results of GO analysis showed that MYBL2 influenced a variety of biological processes, particularly cell proliferation and cancer development. When combined with the results of univariate Cox proportional hazard regression analysis, 3 of 11 genes (*FCGR2A, PDE3A*, and *EPPK1*) were related to the prognosis of patients with melanoma. PDE3A plays an important role in oocyte maturation and vascular smooth muscle cell proliferation (39). Moreover, high PDE3A expression level is associated with many types of tumors (40). EPPK1 is part of the epidermal growth factor (EGF) signaling pathway and promotes cell growth in cervical cancer *via* the p38 signaling pathway (41). At present, there are few studies regarding the *FCGR2A* gene, and its function in cancer is still uncertain. Therefore, our results indicate that *FCGR2A, PDE3A*, and *EPPK1* are the main target genes for MYBL2 and may function as novel cancer biomarkers.

In conclusion, by analyzing the expression and prognostic value of MYBL2 in melanoma through multi-platform data integration, we determined that melanoma has the characteristics of typical MYBL2-dependent tumors. Patients with high MYBL2 expression level suffer a higher risk of recurrence, metastasis, and poorer prognosis. These results suggested that MYBL2 plays important roles in the malignant transformation in melanoma. Moreover, MYBL2 and its downstream transcriptional network can provide effective targets for tumor therapy, and it may be used as a biomarker for the diagnosis and prognosis of melanoma. These findings will provide a reference for the clinical management of melanoma and may lead to further research on the molecular mechanism of melanoma and drug development.

## Data Availability Statement

The datasets presented in this study can be found in online repositories. The names of the repository/repositories and accession number(s) can be found at: NCBI with BioProject PRJNA803358 (https://www.ncbi.nlm.nih.gov/bioproject/PRJNA803358).

## Ethics Statement

The studies involving human participants were reviewed and approved by the Ethics Review Committee of Institute of Tongxu First Hospital (Henan, China). Written informed consent to participate in this study was provided by the participants’ legal guardian/next of kin. The animal study was reviewed and approved by the Ethics Review Committee of Institute of Radiation Medicine, Chinese Academy of Medical Science and Peking Union Medical College (Tianjin, China).

## Author Contributions

BL and TC conceived and supervised the study. BL and FZ designed experiments. FZ and JL performed experiments. BL, FZ, TC, and CG analyzed data. BL, TC, and FZ wrote the manuscript. All authors contributed to the article and approved the submitted version.

## Funding

This work was supported by the Natural Science Foundation of Tianjin city (Grant No. 18JCQNJC13300) and the National Natural Science Foundation of China (Grant Nos. 31861143017 and 81700153).

## Conflict of Interest

The authors declare that the research was conducted in the absence of any commercial or financial relationships that could be construed as a potential conflict of interest.

## Publisher’s Note

All claims expressed in this article are solely those of the authors and do not necessarily represent those of their affiliated organizations, or those of the publisher, the editors and the reviewers. Any product that may be evaluated in this article, or claim that may be made by its manufacturer, is not guaranteed or endorsed by the publisher.
