# Supplementary material for: Downstream Regulatory Network of MYBL2 Mediating Its Oncogenic Role in Melanoma
Source: Front Oncol. 2022 May 18;12:816070. doi: 10.3389/fonc.2022.816070 (PMC9159763; doi:10.3389/fonc.2022.816070)
Supplement: Supplementary file 1 [file DataSheet_1.docx]

**
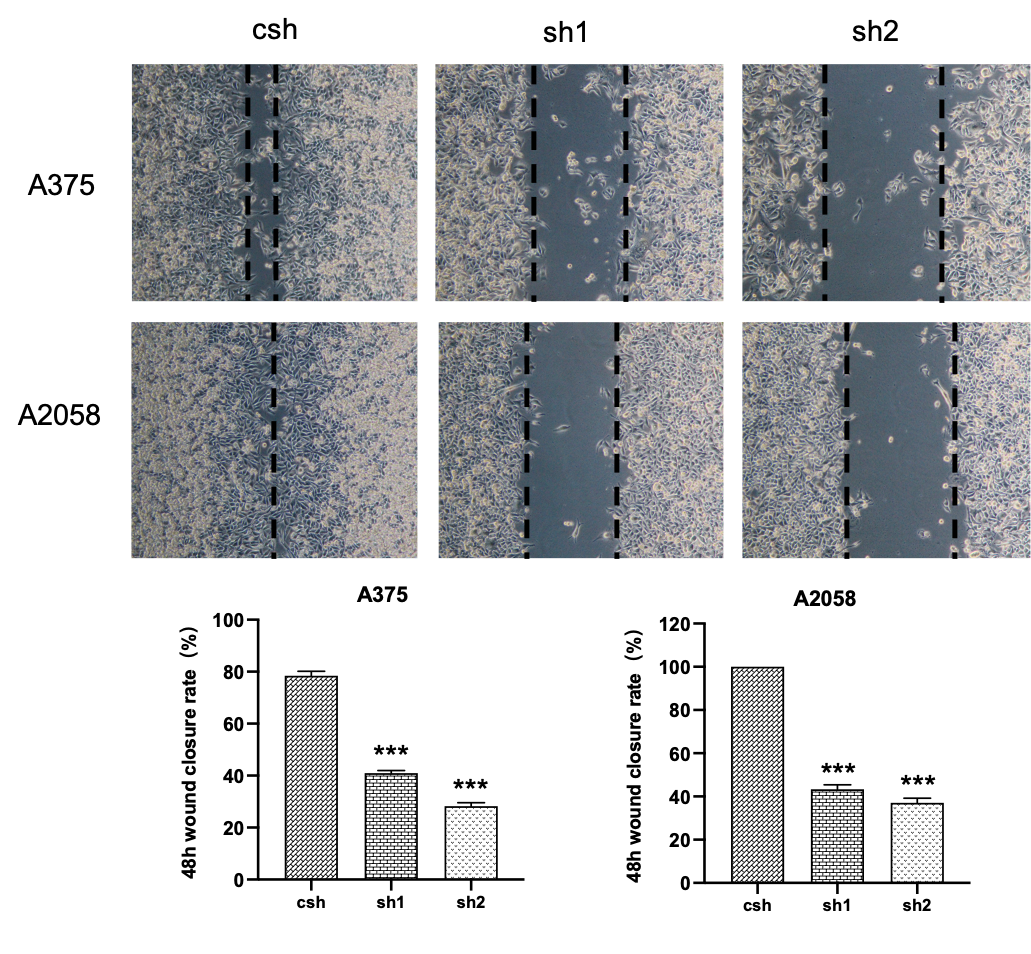
**

**Supplementary Figure 1** Representative bright-field images of the wound healing assay (100×) and corresponding quantitative analysis of the migration closure rates using cells transfected with the indicated vectors at 48 hours post-wounding.

**Supplementary Figure 2** Western blots of MYBL2 in subcutaneous tumor tissues from the shMYBL2 group and csh group.

**
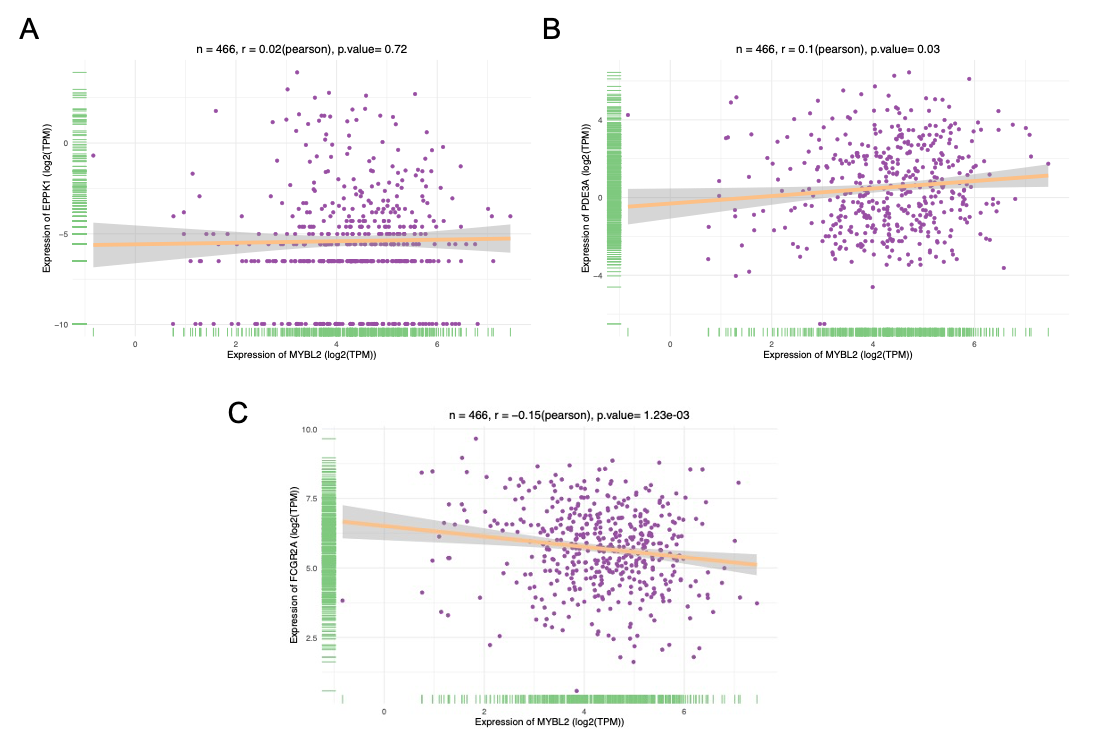
**

**Supplementary Figure 3** Expression correlation diagram of MYBL2 and EPPK1/PDE3A/ FCGR2A through ggstatsplot R package analysis of SKCM data from TCGA dataset.
